# Double spiral chip-embedded micro-trapezoid filters (SMT filters) for the sensitive isolation of CTCs of prostate cancer by spectral detection

**DOI:** 10.1039/d2na00503d

**Published:** 2022-11-04

**Authors:** Hongmei Chen, Qingli Li, Qinghai Hu, Xiaodong Jiao, Wenjie Ren, Shuangshou Wang, Guosheng Peng

**Affiliations:** School of Microelectronics and Data Science, Anhui University of Technology Maanshan 243002 P. R. China; Shanghai Key Laboratory of Multidimensional Information Processing, East China Normal University Shanghai 200241 China qlli@cs.ecnu.edu.cn; School of Chemistry and Chemical Engineering, Anhui University of Technology Maanshan 243002 P. R. China; Department of Medical Oncology, Changzheng Hospital Shanghai 200070 P.R. China

## Abstract

Circulating tumor cells (CTCs) are cancer cells that are released from the original tumor and circulate in the blood vessels, carrying greatly similar constituents as the original tumor. Therefore, CTCs have a significant value in cancer prognosis, early diagnosis, and anti-cancer therapy. However, their rarity and heterogeneity make the isolation of CTCs an arduous task. In the present research, we propose a double spiral chip-embedded micro-trapezoid filter (SMT filter) for the sensitive isolation of the CTCs of prostate cancer by spectral detection. SMT filters were elongated to effectively capture CTCs and this distinctive design was conducive to their isolation and enrichment. The SMT filters were verified with tumor cells and artificial patient blood with a capture efficiency as high as 94% at a flow rate of 1.5 mL h^−1^. As a further validation, the SMT filters were validated in isolating CTCs from 10 prostate cancers and other cancers in 4 mL blood samples. Also, the CTCs tested positive for each patient blood sample, ranging from 83–114 CTCs. Significantly, we advanced hyperspectral imaging to detect the characteristic spectrum of CTCs both captured *in situ* on SMT filters and enriched after isolation. The CTCs could be positively identified by hyperspectral imaging with complete integrity of the cell morphology and an improved characteristic spectrum. This represents a breakthrough in the conventional surface-enhanced Raman scattering (SERS) spectroscopy of nanoparticles. Also, the characteristic spectrum of the CTCs would be highly beneficial for distinguishing the cancer type and accurate for enumerating tumor cells with varied intensities. Furthermore, a novel integrated flower-shaped microfilter was presented with all these aforementioned merits. The success of both the SMT filters and characteristic spectral detection indicated their feasibility for further clinical analysis, the evaluation of cancer therapy, and for potential application.

## Abstract 2

Circulating tumor cells (CTCs) are cancer cells that are released from the original tumor and circulate in the blood vessels, carrying greatly similar constituents as the original tumor.

## Graphical abstract

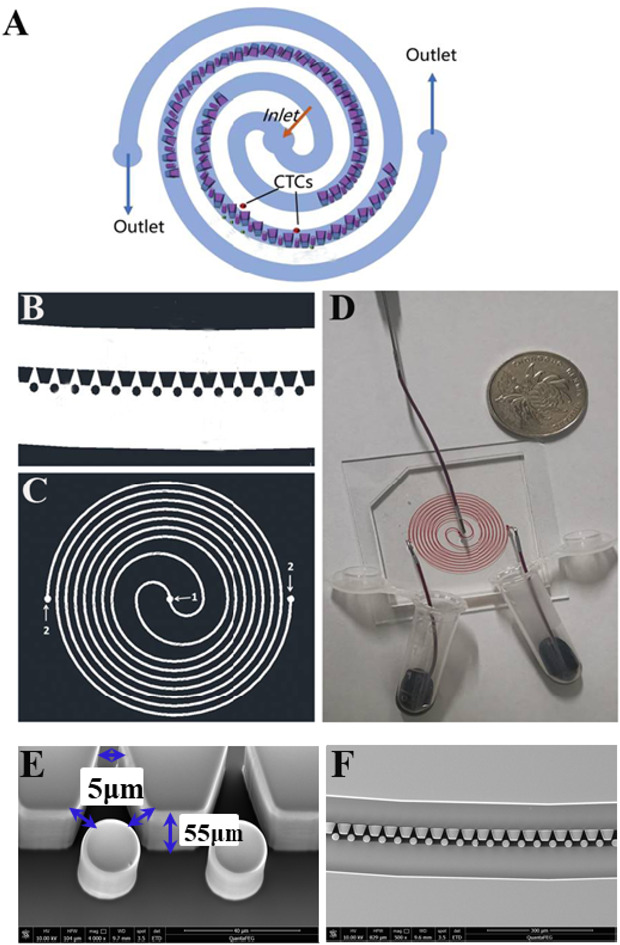

## 1 Introduction

CTCs are tumor cells that are released from the primary tumors and secondary lesions,^1,2^ and move freely in the blood and lymphatic circulation systems. When conditions are mature in a survival environment, a secondary tumor is triggered.^3,4^ This is called the metastasis process and 90% of cancer patients deaths are due to this.^5–8^ Thus, the isolation of CTCs is conducive to understanding cancer and the mechanism of metastasis. However, CTCs are relatively rare: in 1 mL of patient blood, there are only around 1–10 CTCs among 1.0 × 10^7^ white blood cells (WBCs) and 5.0 × 10^9^ red blood cells (RBCs).^7,8^ Therefore, the detection and enumeration of CTCs are difficult to carry out for performing downstream biomolecular genetic analysis. The number of CTCs is related to the disease progress of cancer patients: a lower number of CTCs indicates the disease is in relief, especially after chemotherapy, while a relatively greater number means the disease is becoming worse.^9–13^

Currently, the only clinical application is the Veridex Cellsearch system (Raritan, NJ, USA), which can clinically enumerate CTCs on breast, colon, and prostate and is approved by the US Food and Drug Administration (FDA). It adopts an immunomagnetic approach for CTCs conjugated with immunomagnetic beads. However, it has some disadvantages, such as a capture efficiency no higher than 80% and is only semi-automotive.^14–18^ There is a need to develop a high-efficiency detection system with low cost that is also portable and automatic. Microfluidic chips have attracted attention due to their superiority of small-sized microgaps matching the size of cells, low cost, and low sample consumption. Microfluidic chips are appropriate for isolating CTCs and for *in situ* detection after segregation.

Affinity-based isolation was the earliest approach for isolating CTCs.^19–24^ It needs complex structures to create more collision chances and requires modification by an expensive antibody, such as the anti-epithelial cell adhesion molecule (anti-EpCAM). Small-sized CTCs, low EpCAM-expressed CTCs, and mensenchymal-expressed CTCs tend to flow away. Physical-based isolation^25,26^ could achieve an approximately 90–100% capture efficiency, and a high throughput could be acquired too, especially with a unique circular spiral design.^27–30^ Sarioglu *et al.* presented a chip with triangular microposts arranged in parallel.^29^ Actually, the chip had functioning microfilters for the capture of CTC clusters. Clustering is a rare phenomenon in CTCs' isolation. Also, it was applied with clinical samples of 27 breast cancer patients, 20 melanoma patients, and 13 prostate cancer patients. Preira *et al.* proposed a comb-like microfluidic gradual filter with 24 stages with the width gradually narrowed down from 50 μm to 4 μm.^31^ The channel ceiling heights were 41, 14, and 4 μm for three regions along the flow direction. The comb-like filter was tested with whole blood. Microfilters are good choices for filtering CTCs based on the physical property of their size and deformability. However, the capture purity is restricted in order to enhance the capture efficiency through narrowing down the gaps.

For the circular spiral microfluidic chip,^32–37^ Warkiani *et al.* developed a circular spiral biochip with curvilinear microchannels of 3–4 circles.^34^ The channel was a rectangular cross-section and at the center was the inlet. CTCs were concentrated near the inner wall due to a balance of the inertia lift force and Dean drag force. The sample input flow rate was kept at 100 μL min^−1^. The biochip was characterized with tumor cells spiked into lysed blood. Also, 99.99% of white blood cells (WBCs) were depleted from healthy samples. Furthermore, 7.5 mL red blood cells lysis (RBCL) blood could be processed within 8 min for patients with metastatic breast and lung cancer for a trapezoid cross-section. Lysed blood is blood containing only CTCs and WBCs with red blood cells (RBCs) that have been lysed. After the residue of RBCs in the whole blood has been removed, the only cells left are CTCs and WBCs. As the sizes of tumor cells vary, distinguishing their resolution is not desired alongside leukocytes contamination. Also, clinical samples have complicated issues, such as the number of CTCs, contaminants (WBCs), and quality of blood. Therefore, the purity and wide application of this biochip face relative limitations. Di Carlo *et al.* proposed a passive approach that targets a wide size range of cells by controlling the flow conditions in a single device geometry. The device could generate laminar vortices in lateral cavities that branched out from long rectangular channels.^36^ For spectral detection,^38,39^ surface-enhanced Raman scattering (SERS) has been studied recently.^40–42^ Nanoparticles have been modified to be conjugated with tumor cells. The detection of nanoparticles through their spectral signals allows the detection of tumor cells. The spectral signal can be magnified by having more nanoparticles conjugated on tumor cells. Wu *et al.* proposed a nanoparticle of AuNP–MBA–rBSA–FA,^43^ where MBA is a Raman reporter molecule. The folic acid (FA) on the surface of the AuNP–MBA–rBSA–FA nanoparticles could be distinguished by CTCs of ovarian, brain, kidney, breast, lung, cervical, and nasopharyngeal cancer. There was a linear relationship found between the SERS intensity and concentration of cancer cells in the range of 5–500 cells per mL (*R*^2^ = 0.9935), while the limit of detection was 5 cells per mL and the obvious SERS peak was located at 1076 cm^−1^. Xue *et al.* reported improved SERS-active magnetic nanoparticles of SPION-PEI@AuNPs-MBA-rBSA-FA.^44^ The limit of detection was 1 cell per mL of blood and the linear relationship was used to enumerate CTCs. The numbers of CTCs in the blood of two first-stage clinical patients with cervical cancer were found to be 6 ± 2 cells per 10 mL without SERS-active magnetic nanoparticles and 13 ± 5 cells per 10 mL detected with the SERS-active magnetic nanoparticles.

Hyperspectral imaging as a new approach can be used for CTCs identification, which has never been reported before. Hyperspectral imaging was originally defined by Goetz in the late 1980s and was first discussed for remote sensing of the Earth, as can provide both spatial and spectral information on targets.^45^ The advantage of this technique is that it can obtain a transmit spectrum for each pixel in the image, which can then be used to classify the surface cover materials that cannot be identified with traditional gray or color imaging methods.^46^ In recent years, this imaging technology has been extended to the biomedical engineering field. Microscopic hyperspectral imaging systems have been developed and used to identify lesion areas on tissues. For example, promising results have been found in different studies, including pathological cell segmentation,^47^ tumor detection,^48^ and diseases diagnosis.^49,52^ In cooperation with advanced image processing methods, this technology can also be used for the identification of CTCs.

In the present work, we utilized SMT filters to isolate CTCs from the patient blood of prostate cancer and other cancers. The capture efficiency could reach 94% with tumor cells spiked into phosphate buffer saline (PBS). SMT filters were also validated with artificial patient blood with tumor cells mixed with normal blood. The CTCs tested positive with 10 patient blood samples of prostate cancer and other cancers. The numbers of CTCs were in the range of 83–112 per 4 mL sample. Furthermore, we advanced the detection of CTCs with hyperspectral imaging for both the images and spectra of blood samples for prostate cancer. CTCs were recognized in both ways when *in situ* captured on SMT filters or enriched after capture. There was a peak located at 600 nm for the characteristic spectra recorded.

## 2 Materials and methods

### 2.1 Design of the SMT filters

**Fig. 1:**
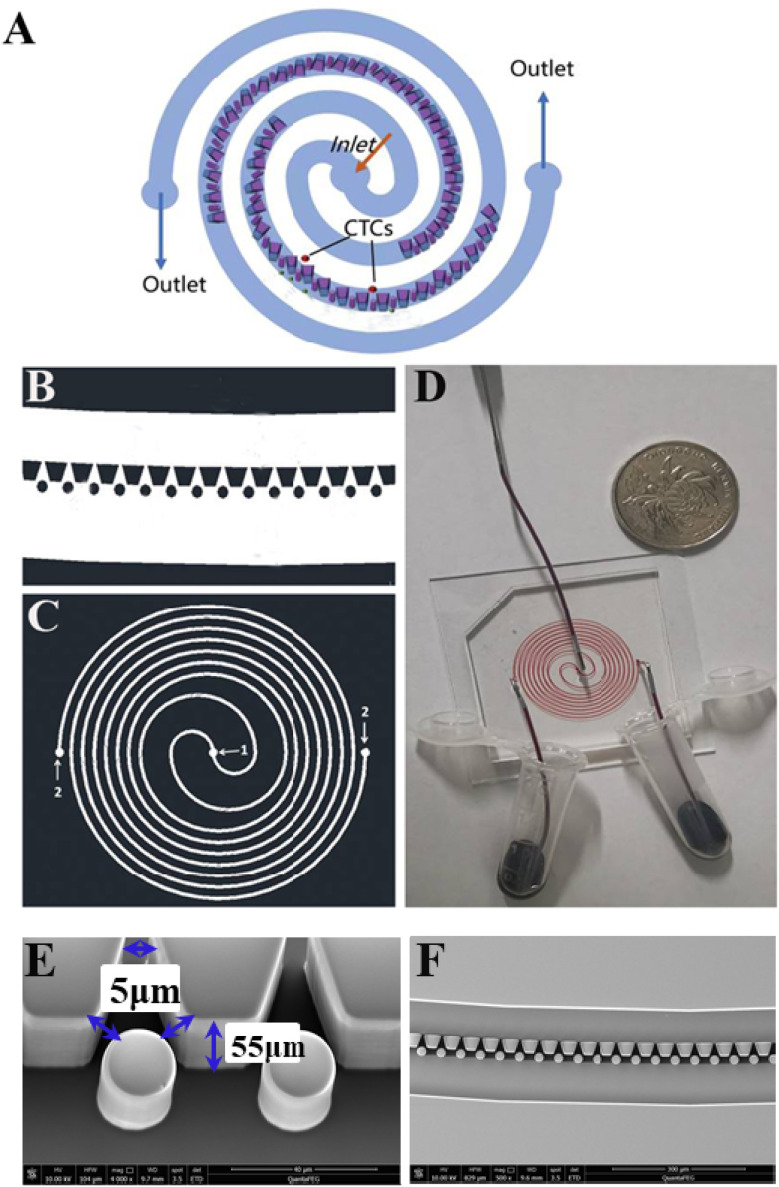
Structure of the SMT filters. (A) Working principle of the SMT filters. CTCs are isolated by micro-trapezoid and circular posts arrays. (B) A small section of the microfluidic chip. (C) Whole structure of the SMT filters. (D) Clinical assays of patient blood samples were segregated by SMT filters. (E) Scanning electron microscopy image of the trapezoid-spiral filter, showing the sizes of the gaps and height. (F) Arrays of trapezoidal and circular microstructures set in the middle of the microtunnel.

The SMT filters consisted of two circular spiral microchannels surrounding each other. As shown in Fig. 1, there were two arrays of trapezoid and circular microposts embedded inside the channels. These two arrays of posts formed a barrier to allow them to function as microfilters to capture CTCs. The two closest points of two trapezoid posts formed a capture gap of 5 μm. Also, the same gap distance of two capture sites was for the two shortest points of the trapezoid and circular posts. When whole patient blood was introduced, the CTCs were subject to 2–3 times capture in 5 μm capture sites. Close to the center, the posts arrays were connected with the outside wall of the channel. Also, approaching the end of the filters, the micropost arrays were joined with the inside wall of the channel. This design meant the whole patient blood had to pass through the microposts barrier in order to flow out, which was also convenient for enrichment. Conventionally the capture length of the posts barriers of the microfilter were approximately 2–3 cm. However, the capture length for the SMT filters was over 20 cm of double 3–4 circles, which effectively ensured a high capture efficiency. When patient blood was introduced from the center, it could flow highly along the two circular microchannels. For enrichment, flushing reversely, the captured CTCs could be collected. Fig. 1D shows clinical patient assays with whole patient blood samples. The whole patient blood samples were spiked from the center, and after capture, the blood cells were gathered.

The inertial lift force was used to converge the randomly distributed particles to a single streamline at high flow rates. Drag and lift forces play a vital role for particle movement in fluids. Two Reynolds numbers describe the flow of particles in closed channel systems: the channel's Reynolds number (*R*_c_) and the particles' Reynolds number (*R*_p_),^33^1
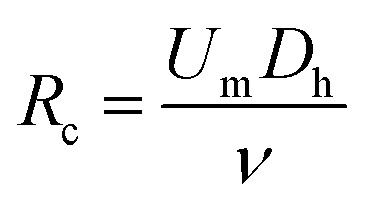
2
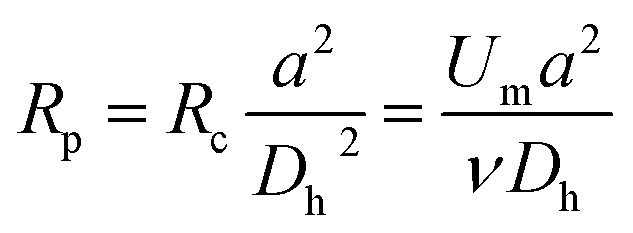
where *U*_m_ is the kinematic viscosity of the fluid, *ν* = *μ*/*ρ* (where *μ* and *ρ* are the dynamic viscosity and density of the fluid, respectively), and *D*_h_ is the hydraulic diameter, defined as 2*wh*/(*w* + *h*) (where *w* and *h* are the width and height of the channel).

Particle behavior is dominated by the inertial lift forces when the particle Reynolds number is of order 1. The magnitude of the lift forces (*F*_z_) in a parabolic flow is given by the following equation,3
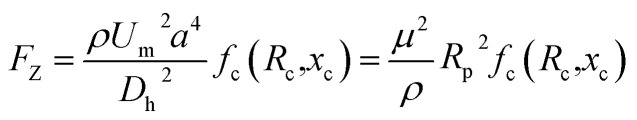
where *f*_c_(*R*_c_,*x*_c_)is a lift coefficient. For the equilibrium position, the wall effect is balanced by the shear-gradient lift, *f*_c_ = 0.

The inertial lift force on a particle leads to migration away from the channel center. An expression for the particle migration velocity, *U*_P_, can be deduced assuming Stokes drag,*F*_s_ = 3π*μaU*_P_where *U*_P_ balances the lift force.4
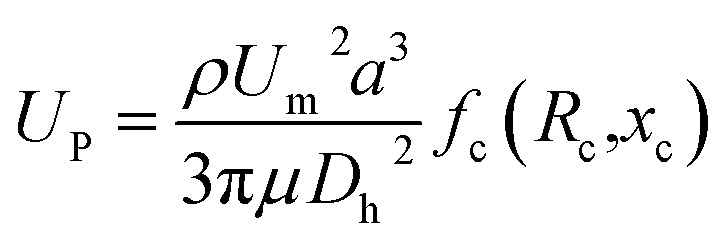


Secondary rotational flow can change the position of flowing particles, which is called the Dean flow. The Dean flow is composed of two counterrotating vortices with flow directed toward the outer bend at the midline of the channel and inwards at the channel edges. The drag attributable to Dean flow (Dean drag, FD) is as FD ∼ *ρU*_m_^2^*aD*_h_^2^*r*^−1^. The preferred location of particles in channels with a curved geometry is determined by the balance between the inertial lift and Dean drag forces. In our situations, the trapezoid and circular arrays functioned as a capture barrier of the filter. The filter lay in the middle of the circular microchannel. The starting section was connected with the outer wall of the channel and the ending section was joined with the inner wall. When whole patient blood was introduced from the center, it moved in front of the microposts barrier. This barrier blocks CTCs with microposts and capture sites. In order to move out, it has to traverse the microposts barrier. After crossing the barrier filter, blood cells smoothly move out from the other separated parts of the circular microchannels. In this case, there is no need to consider the effects of the inertial lift and Dean drag forces.

### 2.2 Fabrication

The chip was fabricated by soft lithography. The patterns of the microstructure were drawn on to produce a high-resolution transparent optical photomask. The silicon wafer was spin-coated with a 7 μm-thick AR-N 4450-10 (ALLRESIST GmbH, Germany). After soft baking, the wafer was exposed to UV light and then post-exposure baked. After developing, a silicon master pattern with the microstructure was generated. The height of the microposts or thickness of the chip was 55 μm. By casting liquid polydimethylsiloxane (PDMS) (a 10 : 1 mixture of the base and curing agent, Sylgard 184, Dow Corning Inc.) against the master and baking in an oven for 1 h at 80 °C, a PDMS structure was fabricated with the inlet and outlet (1.0 mm) punched. Next the structure was treated with a high-frequency generator (Electro-Technic Products, Inc., Chicago, IL), and the PDMS structure was bonded to a glass slide after thorough ultrasonic cleaning.^50^

### 2.3 Cell culture

MCF-7 cells and MDA-MB-231 cells (human breast adenocarcinoma) were provided by Nanjing University, and HeLa cells were offered by Peking University Third Hospital. The cells were cultured in Dulbecco's modified Eagle medium (DMEM) (HyClone, USA) supplemented with 10% fetal bovine serum (FBS) (GIBCO, USA) and 1% penicillin–streptomycin (Ying Reliable Biotechnology, China), and incubated in a humidified atmosphere at 37 °C with a 5% CO_2_ atmosphere. When the cell lines had grown as adherent monolayers to 95% confluence, they were detached from the culture dishes with 0.25% trypsin solution for 2 min.

### 2.4 Staining

Calcein AM (BIOTIUM, USA) was used to stain all the MCF-7 cells, and Hoechst (Life, USA) was used to stain the DNA in all the cell nuclei. Labeling was completed by two approaches: one, by putting the staining reagents into the assay directly, and the other by putting into the cell suspension. Next, 1 mL of 1× PBS containing 10 mg mL^−1^ (1%) bovine serum albumin (BSA, Solarbio, China) and 0.05% Tween-20 was used with cancer cells to reduce the non-specific cell adhesion on the surface of the structure. For cells captured on the Hoechst chip, Cytokeratin-FITC (BD Biosciences) and CD45-PE (BD Biosciences) were used for on-chip staining. A small limited number of unlabeled tumor cells were spiked into the lysed and whole blood. Those artificial patient bloods were processed through the chip followed by washing with PBS, fixing, and permeabilization. Anti-cytokeratin (BD Biosciences) and anti-CD45 (BD Biosciences) and Hoechst applied in 1% bovine serum albumin were utilized for all the samples. After washing, then the samples were ready for microscopic imaging.^45^

### 2.5 Hyperspectral imaging

**Fig. 2:**
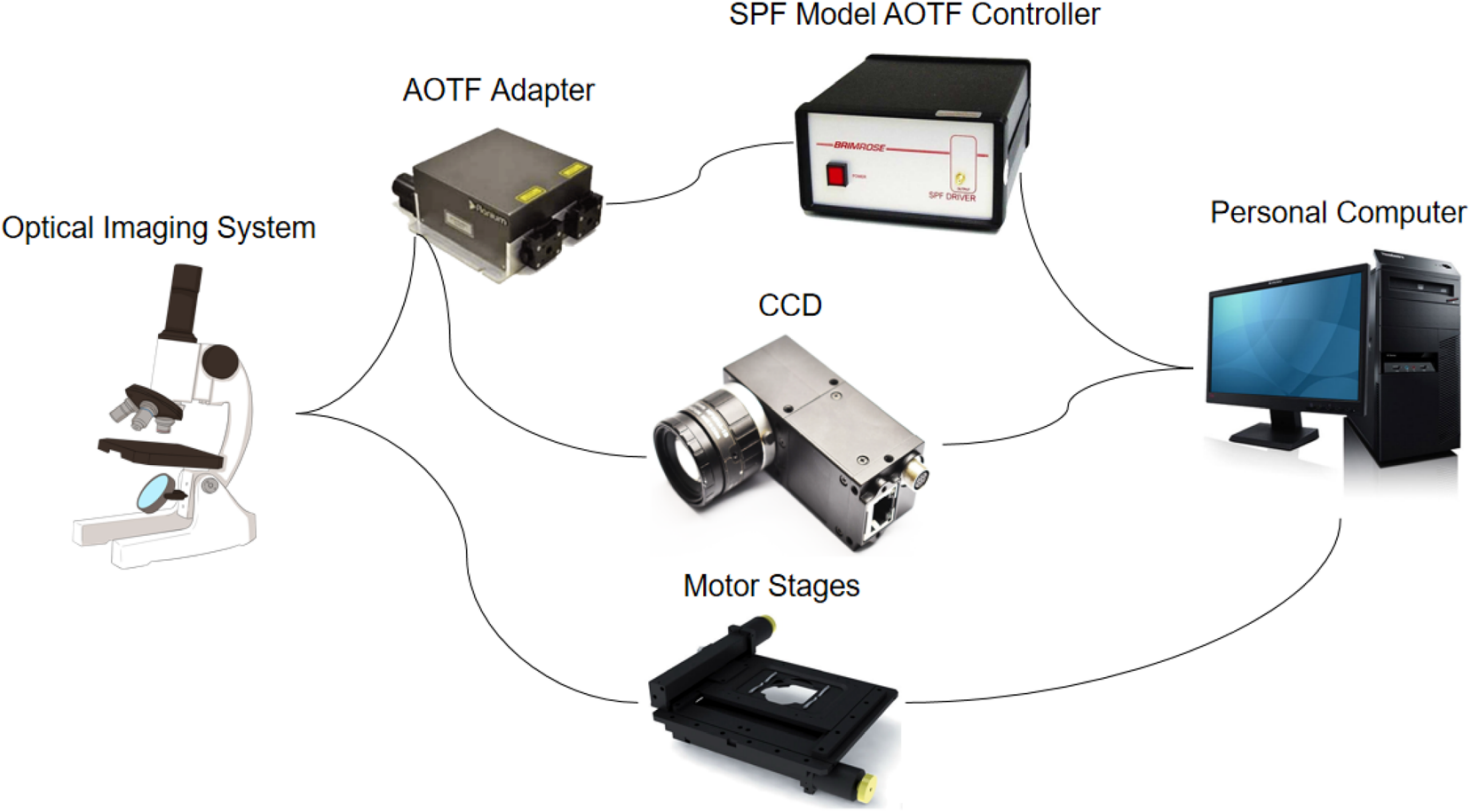
Microscopic hyperspectral imaging system.

The microscopic hyperspectral imaging system used in this paper was developed by our group. The system is shown in Fig. 2. It consisted of an optical imaging system, an AOTF adapter, an SPF Model AOTF controller, a high-density cooled charge-coupled device detector (CCD), and a personal computer. The optical imaging system was mainly composed of four parts: an optical microscope, a ring light source, a high-precision three-dimensional electric stage, and a shockproof stage. AOTF is an electro-optical modulation device, which controls the diffraction of incident narrow-band light by controlling the RF frequency applied to the AOTF, and the intensity of diffracted light transmitted through AOTF can be adjusted accurately and quickly by changing the power of the RF signal. Its spectral resolution is very high in some ranges, and there are no mechanical moving parts. It has a fast wavelength adjustment speed and high flexibility. It is relatively easy to integrate with some existing optical detection equipment in medicine, such as microscopes and endoscopes. Hyperspectral images can be taken with this device. The SPF AOTF Controller is a high-performance RF frequency generator. It provides a fast frequency sweep using a direct digital synthesizer incorporated into a self-contained case with an AC power supply. The CCD is a charge-coupled device, which is a detection element that expresses the signal size by the amount of charge and transmits the signal by coupling. The spectral angle (SAM) method should be changed to the spectral angular mapper (SAM) method. The spectral angle mapper (SAM) is a spectral matching technique that can distinguish the spectral curve of each pixel point based on the similarity between the estimated pixel spectrum and the sample spectrum or the sub-pixel end member spectrum of the mixed pixel.^51^

The chip with the cells was placed on the stage of the microscopic hyperspectral imaging system, and then the hyperspectral images could be captured. The spectral range of the system was 450–750 nm, which included 40 single bands. At the same time, the color images of the same field of view were also collected to facilitate subsequent processing and comparison. Each chip selected the 25–30 most representative fields of view for capturing. First, the image framed the region of the cell. For this region, the spectral curve of each pixel was averaged to obtain the average spectral curve and spectral characteristics representing the whole cell region. Then, the K-means method for binary classification tasks and spectral angle (SAM) method were used to obtain the complete structures of the cells. Combining the spectral information and spatial information from the hyperspectral images, the type of cancer could be identified.

### 2.6 Simulation

Through Comsol simulation, the pressure field, velocity field, and streamline behaviors and influence on the following SMT filters could be determined. The software parameters were set at 1054 kg m^−3^ for the liquid density, 4 × 10^−3^ Pa s for the dynamic viscosity, and the velocity of the inlet was 2.778 × 10^−10^ m^3^ s^−1^.

## 3 Results and discussion

### 3.1 Capture efficiency of the SMT filters with spiked tumor cells

**Fig. 3:**
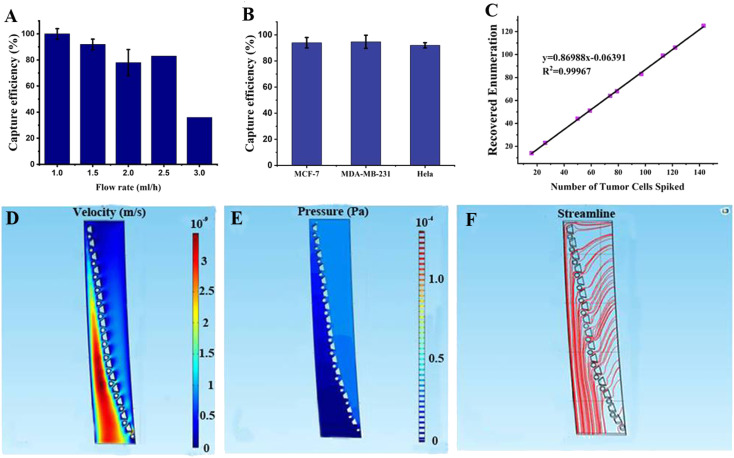
Capture efficiencies of CTCs with various cell types and cell numbers and the simulation analysis. (A) Capture efficiency for the trapezoid-spiral chip at different flow rates. The capture efficiency could reach 90% at 1.5 mL h^−1^ and decrease down to 30% at 3 mL h^−1^. (B) Capture efficiencies for three different cell lines of breast (MCF-7), breast (MDA-MB-231), and HeLa cells, respectively (*n* = 3). (C) Capture efficiency for varied cell numbers ranging from 10 to 100. (D) Simulation of the velocity field (m s^−1^), (E) Pressure field simulation (Pa). (F) Simulation of the streamlines of fluidic flow for a small section of the microposts array of SMT filters, respectively.

As shown in Fig. 3, the capture efficiency was determined at different flow rates and the optimal flow rate was determined to be 91% at 1.5 mL h^−1^. Three cancer cell lines were used to validate the capture efficiency of the SMT filters: MCF-7, MDA-MB-231, and HeLa, respectively. Here, MCF-7 cells are human breast cancer cells with the highly expressed epithelial cell adhesion molecule (EpCAM); MDA-MB-231 cells were also human breast cancer cells with lowly expressed EpCAM, and HeLa is a HeLa cell cervical cancer model with lowly expressed EpCAM. With these three cancer cell lines, SMT filters could be validated with three assays for each one to obtain statistical data of the capture efficiency. The optimal flow rate was set at 1.5 mL h^−1^. As shown in Fig. 3, the capture efficiencies for the MCF7, MDA-MB-231, and HeLa cells were 94%, 95%, and 93%, respectively. For 10–100 tumor cells of MDA-MB-231, there was a linear relationship between the captured and spiked cell samples with a slope of 87%.

### 3.2 Simulation of the SMT filters

We choose a section of an array of SMT filters for the fluidic simulation in Fig. 3. The top was the inlet, and the sample flowed from the right side of the microposts barrier to the left. When the fluid approached the microposts, it traversed the barrier and flowed out from the other side. The velocity was low on the right side of the posts array and increased dramatically in the other region below the post barrier. In this region, the flow velocity tended to increase relatively with the ongoing flow. The reason is that the patient blood sample approaches slowly to the post barrier and flow extremely fast in order to move out from the other side. This indicated that the CTCs tended to move slowly to the post barriers and were captured there, while hematological cells were dramatically squeezed out and were depleted rapidly. For pressure behavior analysis of the simulation, the pressure was high above the post array and then reduced to a low value after traversing the barrier. This illustrated that the patient blood samples experienced high pressure and were pushed directly toward the microposts array. After the CTCs were captured by the microposts, they would not be pushed out due to a sudden drop of pressure, inducing a small force on the captured CTCs over the other side of the posts connected to the outlet. This change ensured a high capture efficiency, while high depletion produced high viability. For the streamline tendency, the continuous streamlines went curvedly toward the post array, and then removed straightly. This means the patient blood samples flowed through the trapezoid-circular posts arrays. The CTCs would move toward the posts barrier and be captured there, while the blood cells would go through the gaps in the barrier and flow out tremendously and directly.

### 3.3 Clinical assays of patient blood samples of prostate and other cancers

**Fig. 4:**
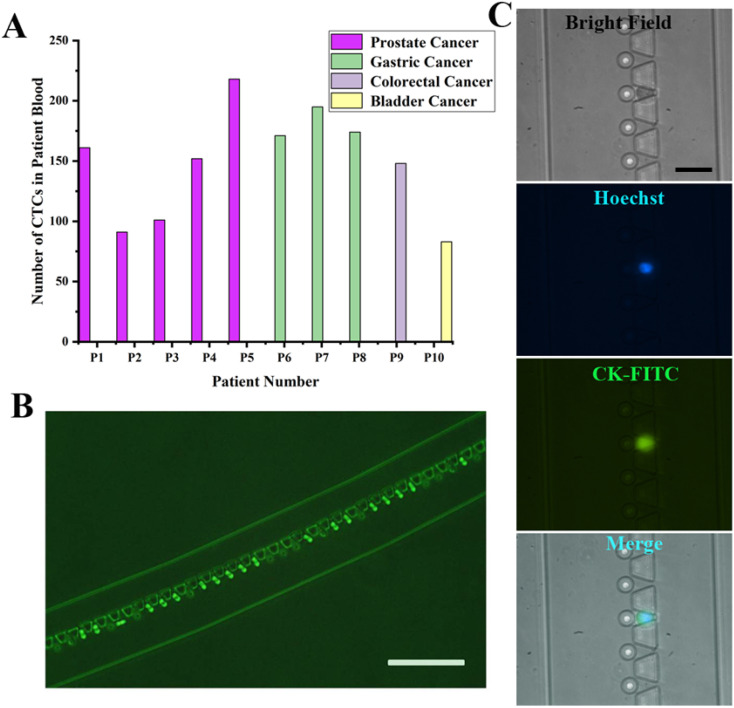
Clinical evaluations of the SMT filters with patient blood samples of prostate and other cancers. (A) Clinical performance of the SMT filters showing the number of CTCs detected from patients with prostate and other cancers. (B) Immunofluorescence images for CTCs detected from prostate cancer of CK-FITC (scale bar: 200 μm). (C) Immunofluorescence images for CTCs detected for bright field, Hoechst, CK-FITC, and merged cells (scale bar: 50 μm).

We tested 10 patient blood samples to verify the efficacy of the SMT filters: 6 samples came from prostate cancer, 2 from gastric cancer, 1 from colorectal cancer, and 1 from bladder cancer. The CTCs tested positive for all the patient blood samples, ranging from 73 to 218 for each 4 mL patient blood sample. The CTCs could be identified with Hoechst+/CK+/CD45-, recognized with blue and green fluorescence. For the *in situ* images of CTCs captured on the SMT filters, no WBCs appeared, indicating the capture purity could reach over 90%. The patient numbers for the SMT filters evaluation corresponding to the number of CTCs detected are illustrated in Fig. 4. A green immunofluorescence image could be obviously observed for the CK-FITC for CTCs of prostate cancer *in situ* captured on the filters. It was demonstrated that the SMT filters could successfully isolate and discriminate CTCs from prostate and other cancers.

Also, releasing the captured CTCs from the device was an easy and fast operation. Due to the unique design of the circular spiral structure embedded with trapezoid microposts, the microposts barrier was connected with the outside wall of the microchannel at the beginning of the inlet and the inner wall at the outlet. During the capturing process, the whole patient blood has to pass through the barrier in order to be captured. The CTCs were captured at the capture sites formed by trapezoid and circular microposts. When reversely flushing with PBS rapidly from the outlet in a few minutes, the captured CTCs could be released from the device. The flow rate was as high as about 2 mL min^−1^, and the time for passing the PBS was 2 min to completely enrich the captured CTCs. Since most captured CTCs were not in the captured sites and were just in front of the trapezoid microposts barrier, the release was simple and protective. Therefore, this was a non-destructive approach to enrich the captured CTCs.

### 3.4 CTCs isolation of clinical patient samples with spectral detection

**Fig. 5:**
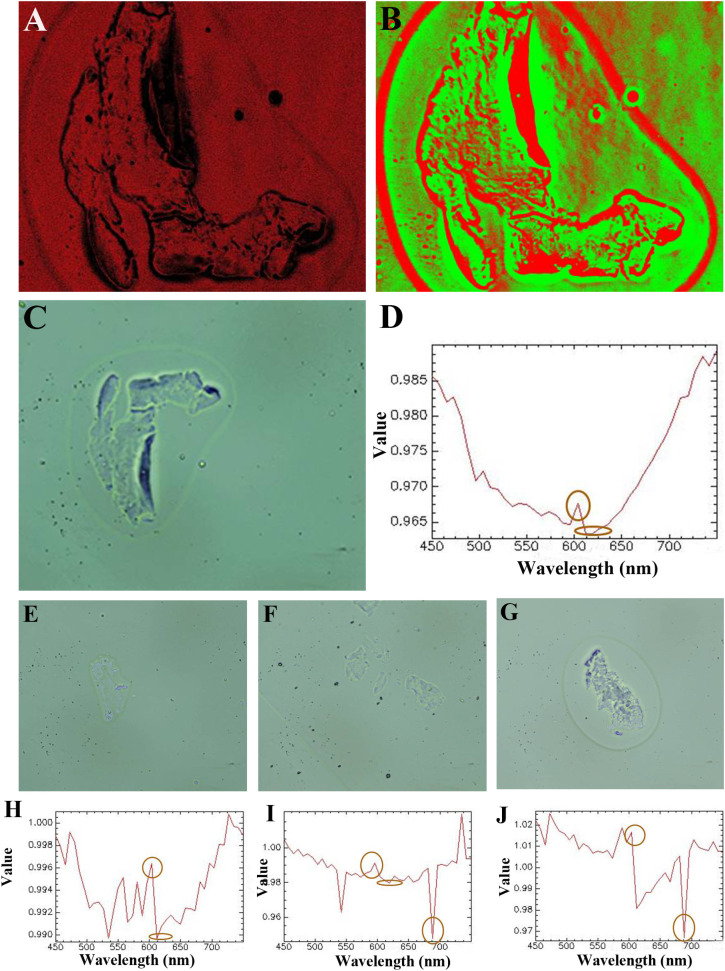
Hyperspectral characterizations of CTCs of prostate cancer. (A) Colorful hyperspectral image of a CTC captured from prostate cancer showing the complete structure. (B) Colorful hyperspectral image of a CTC captured from prostate cancer showing the complete structure. (C) Hyperspectral image of another CTC captured from prostate cancer with the complete morphology of the cell membrane and cytoplasm. (D) Hyperspectral spectrum of CTC isolated with an obvious peak located at 600 nm for CTCs captured in (A)–(C). (E) Hyperspectral image of a CTC captured from prostate cancer. (F) Hyperspectral image of a CTC captured from prostate cancer. (G) Hyperspectral image of another CTC captured from prostate cancer. (H) Hyperspectral spectrum of a CTC isolated with an obvious peak located at 600 nm for the CTC captured in (E). (I) Hyperspectral spectrum of CTC isolated with an obvious peak located at 600 nm for the CTC captured in (F). (J) Hyperspectral spectrum of a CTC isolated with an obvious peak located at 600 nm for the CTC captured in (G).

The advanced evaluation and identification of CTCs were performed by characteristic spectral detection with hyperspectral imaging. Patient blood samples came from three prostate cancers and one bladder cancer. We performed *in situ* staining on SMT filters first to distinguish the CTCs captured, and then the captured CTCs could be recognized with hyperspectral imaging for characteristic spectral detection. The CTCs samples were from CTCs captured *in situ* on SMT filters or enriched after the isolation. As shown in Fig. 5, the cancer morphology could be clearly distinguished in the cell membrane and cytoplasm by the hyperspectral images. The CTCs could be characterized from the morphology, and in this was a doctor could gain bimolecular genetic information from both the hyperspectral image and characteristic spectrum. Compared with SERS, the spectrum recorded is the signal released from the nanoparticles and antibodies from modification on them, rather than the characteristic spectrum of the tumor cells themselves. Also, the spectra from clinical patient samples are usually obscure with much interference. However, the hyperspectral spectrum can detects the characteristic spectrum of a sample directly emitted from the tumor cells itself. Also, from Fig. 5, it could be seen that the characteristic spectrum was very clear with a peak located at approximately 600 nm indicating high purity. The formant at 600 nm shown in Fig. 5 was mainly the contribution from the captured cancer cells, while the contribution of the background was relatively small, because there was no obvious peak at 600 nm in the background and spectral characteristic curves of other non-cancer cells. This latter point also corresponded to the findings in other literature studies, where the spectral characteristics of the captured tumor cells were reported to show an obvious peak at 600 nm.^47^

Since the spectrum is emitted from the CTCs themselves, it could be used to identify the tumor cell type by the specific peak positions located. Furthermore, if the fitting line of intensity corresponding to the number of tumor cells could be determined, CTCs enumeration could be identified from the fitting line. This was further carried out in our next step.

Due to there being some coincident sizes of CTCs and leukocytes, there would be some leukocytes occasionally captured on the device. However, the physical property differences of CTCs and leukocytes are their size and deformability. Leukocytes are more deformable than CTCs. With whole patient blood, the sample would flow through the device at a flow rate of 1.5 mL h^−1^. The chip was red with the contamination of WBCs and RBCs after capture. Then, we flowed 1.0 mL PBS through the chip at the same flow rate. Since the flow rate did not change, this would not affect the capture and the captured CTCs would not flow away. Most captured CTCs were captured in front of the trapezoid microposts barrier. After 40 min, the WBCs and RBCs on the chip could be flushed away from the chip completely. RBCs are smaller and could pass through the microposts barrier easily, and the same for most small-sized WBCs. For some WBCs with a coincident size of small CTCs, they are easily deformable and can pass through the trapezoid microposts barrier. However, here a high capture purity could be seen from the spectrum, since there was only one single spectral line shown on the graph without any disturbance. This indicated the capture purity was very high for the capture in this device.

## 4 Discussion

The SMT filters showed good performance in the isolation of CTCs of prostate cancer with high efficiency. There were two arrays of trapezoid and circular posts organized as a capture barrier. Usually for circular spiral microfluidic chips, the equilibrium positions of the particles are governed by combined two forces: shear-induced lift force (*F*_s_) and sustaining wall lift force (*F*_w_). Under the influence of these two forces, migrating particles approximately equilibrate at 0.2*D* away from the wall with *D* being the diameter of the channel. This influence was not considered for the situation of the SMT filters, since the micropost barriers were arranged to function as microfilters. CTCs were seized at the few capture sites formed and the blood cells flowed away smoothly with the unique design. However, for the SMT filters, the capture length was elongated to around 20 cm. The throughput was not as high as in a conventional circular spiral chip without any microposts embedded. With 7.5 mL blood after RBCL, the process only took 8 min for a trapezoid cross-section. The flow rate for the SMT filters could reach 1.5 mL h^−1^. The solution could be used to shorten the capture length for the remaining 2–3 circles, since there was only 1–10 CTCs in 7.5 mL whole blood.

In this work, we employed both hyperspectral images and the spectra to distinguish CTCs from prostate cancer. From the images taken, the integrity of the CTCs captured for the cell membrane and cytoplasm could be observed. The information gathered from observing the complete morphology was richer than in traditional immunofluorescence images. Also, for the hyperspectral spectra, obviously there was only one clear curve without any interference of the aniline impurity. The spectrum recorded was from CTCs of prostate cancer and thus showed the characteristic spectrum of the tumor cells themselves. Instead, the magnified SERS signals were from the nanoparticles conjugated on tumor cells. From these four hyperspectral spectra recorded, it could be seen that there was an obvious peak located at 600 nm for this prostate cancer. This also illustrated that the capture purity of the SMT filters was very high. With observing particular peak positions, the cancer type could be identified. However, the composition of patient blood is always very complicated.

## 5 Novel design of an integrated flower-shaped microfluidic chip

**Fig. 6:**
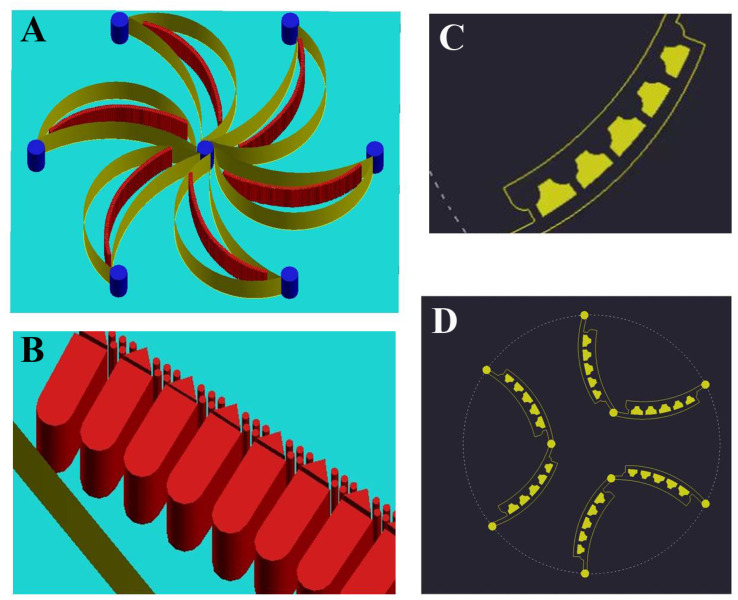
New design of an integrated flower-shaped microfluidic chip of two layers: (A) a flower-shaped microfluidic chip with six petals embedded with six arrays of microfilters. (B) Microposts of microfilters composed of rectangular, triangle, and circular microposts; (C) bowl-shaped microfilters in the second layer; (D) six microfilters arranged in the second layer with bowl-shaped cavities to trap CTCs.

Based on the discussed performance above of the SMT filters, we proposed a flower-shaped microfluidic chip in Fig. 6. The chip was composed of six channels in the shape of petals. Inside the petals there were microfilters arranged. The microposts consisted of half circular, rectangular, triangle, and circular ones, organized in arrays as microfilters. The microposts arrays were inclined, convenient for enrichment with flushing in reverse. For the second layer, the microposts were arranged to form concave bowl-shaped cavities to trap CTCs. Also, the blood cells flowed away. The six outlets from the first layer comprised the six inlets of the second layer. This unique design ensured a high capture efficiency and favors spectral detection.

## 6 Conclusion

Highly efficient unique SMT filters were studied and their efficacy validated with tumor cells and whole patient blood. The capture efficiency could reach 94% and the capture purity could be over 90%. In the validation, 10 clinical patient blood samples were used to validate the SMT filters with fluorescence recognition of the CTCs of prostate and other cancers. The CTCs tested positive for each patient sample, ranging from 73–218 CTCs. Furthermore, both hyperspectral imaging and spectroscopy techniques were developed for detecting CTCs in 4 clinical patient blood samples. The complete morphologies of the cell membrane and cytoplasm could be characterized with integrity. Clear characteristic spectra were obtained with a peak located at 600 nm. Next, we found linear relationship between the intensity and number of tumor cells. From this linear fit, the number of CTCs could be identified after separation from the microfluidic chips. Also, the characteristic spectrum from the hyperspectral spectrum could be utilized to determine the tumor type. An advanced, integrated flower-shaped microfilter is thus presented to satisfy high capture efficiency and high purity. Both the success of SMT isolation and spectral detection capability facilitate its further use for clinical assays, cancer therapy, and potential medical applications.

## Ethical statement

All experiments were performed in compliance with relevant laws or guidelines. All experiments followed institutional guidelines of East China Normal University, Anhui University of Technology and Changzheng Hospital.

The institutional committee(s) of East China Normal University, Anhui University of Technology and Changzheng Hospital that approved the experiments; patient blood samples were supplied by the Fourth Affiliated Hospital of Anhui Medical University under approval.

## Conflicts of interest

The authors declare that they have no conflict of interest. The manuscript is approved by all authors for publication.

## Supplementary Material
